# Delayed ^18^F-FDG PET imaging provides better metabolic asymmetry in potential epileptogenic zone in temporal lobe epilepsy

**DOI:** 10.3389/fmed.2023.1180541

**Published:** 2023-07-03

**Authors:** Yang Hong, Chang Fu, Yazhou Xing, James Tao, Ting Zhao, Na Wang, Yanan Chen, Yang You, Zhe Ren, Yingxing Hong, Qi Wang, Yibo Zhao, Yang Yang, Jiewen Zhang, Junling Xu, Xiong Han

**Affiliations:** ^1^Department of Neurology, People’s Hospital of Henan University, Henan Provincial People’s Hospital, Zhengzhou, Henan, China; ^2^Department of Nuclear Medicine, People’s Hospital of Zhengzhou University, Henan Provincial People’s Hospital, Zhengzhou, Henan, China; ^3^Department of Neurosurgery, People’s Hospital of Zhengzhou University, Henan Provincial People’s Hospital, Zhengzhou, Henan, China; ^4^Division of Biological Sciences, University of Chicago, Chicago, IL, United States; ^5^Department of Neurology, People’s Hospital of Zhengzhou University, Henan Provincial People’s Hospital, Zhengzhou, Henan, China; ^6^Beijing United Imaging Research Institute of Intelligent Imaging, Beijing, China

**Keywords:** ^18^F-FDG PET/CT, temporal lobe epilepsy, asymmetry index, two time point imaging, potential epileptogenic zone

## Abstract

**Objective:**

To investigate the value of ^18^F-FDG positron emission tomography/computed tomography (PET/CT) two time point imaging for the identification of the potential epileptogenic zone (EZ) in temporal lobe epilepsy (TLE).

**Methods:**

Fifty-two patients with TLE were prospectively enrolled in the ^18^F-FDG PET/CT two time point imaging study. The early imaging was obtained approximately 40 min (43.44 ± 18.04 min) after ^18^F-FDG injection, and the delayed imaging was obtained about 2 to 3 h (160.46 ± 28.70 min) after the injection. Visual and semi-quantitative analysis of ^18^F-FDG uptake were performed at the two time points in EZ and contralateral symmetrical region. The mean standardized uptake value (SUVmean) of EZ and contralateral symmetrical region was calculated to determine the asymmetry index (AI) of the early and delayed images, as well as in the MRI positive and negative patient groups.

**Results:**

Semi-quantitative analysis demonstrated that AI of the early and delayed ^18^F-FDG PET/CT images was 13.47 ± 6.10 and 16.43 ± 6.66, respectively. The ΔAI was 2.95 ± 3.05 in 52 TLE patients between the two time points. The AI of the EZ was significantly elevated in delayed images compared to the early images (*p* < 0.001). The AI of delayed imaging was also significantly elevated compared to the early imaging in both MRI positive (ΔAI = 2.81 ± 2.54, *p* < 0.001) and MRI negative (ΔAI = 3.21 ± 3.91, *p* < 0.003) groups, and more pronounced in MRI negative group. Visual analysis also showed that the delayed imaging appeared to be superior to the early imaging for identification of potential EZ.

**Conclusion:**

Delayed ^18^F-FDG PET imaging provided significantly better than the early imaging in the identification of potential EZ, which can be valuable during epilepsy pre-surgical evaluation in patients with TLE.

## Introduction

Epilepsy is the second most common neurological disorder, of which 60–70% are temporal lobe epilepsy (TLE) (1). Although seizures in most patients are controlled with anti-seizure medications (ASMs), 30–40% patients will develop drug resistant epilepsy despite the development of new generations of ASMs. Surgical removal of epileptogenic zone (EZ) is the most effective treatment for patients with drug resistant epilepsy. There is a significant correlation between surgical outcomes and accuracy of EZ identification (2). Electroencephalography (EEG), magnetic resonance imaging (MRI) and ^18^F-deoxyglucose positron emission tomography (^18^F-FDG PET) have been widely used for the localization of EZ (3). The advent of new techniques such as magnetic resonance imaging spectroscopy (MRS), magnetoencephalography (MEG), and multimodal magnetic resonance imaging has further improved the accuracy for the localization of EZ. However, these new modalities have relatively limited clinical applications due to their long sequence scanning time and high costs.

The precise localization of EZ remains a major challenge. Studies have shown that^18^F-FDG PET plays an important role in preoperative evaluation and can provide lateralizing or localizing information in 60–90% of patients with TLE (4, 5). ^18^F-FDG PET can detect areas of the brain with reduced FDG uptake (glucose hypometabolism) in epileptogenic foci. It is particularly valuable when scalp EEG provide inadequate data for the localization, and MRI is normal (3). In MRI negative and PET positive cases, ^18^F-FDG PET images can be valuable to guide the electrode placement during intracranial EEG monitoring, leading to favorable surgical outcomes (6).

^18^F-FDG PET two time point imaging technique has been commonly used for the diagnosis and classification of both benign and malignant tumors, as well as for the detection of tumor recurrence (7, 8). However, it has been rarely used for the diagnosis and localization of epileptic seizures. Recently, Liu et al. performed PET/MRI two time point imaging in 41 patients with epilepsy including 10 patients with TLE. They concluded that delayed imaging was superior to early imaging in identifying metabolic asymmetries between the potential EZs and the contralateral regions (9). As temporal lobe epilepsy is the most common of the epilepsy, in this study we focused only on temporal lobe epilepsy.

The purpose of this study was to compare the effectiveness of ^18^F-FDG PET two time points imaging for the identification of potential epileptogenic zone in temporal lobe epilepsy.

## Methods

### Patients

Fifty-two patients were prospectively enrolled in the study from June 2021 to February 2023 in Henan Provincial People’s Hospital (Table 1). There were 27 male patients (51.92%), and the mean age was 29.54 ± 13.09 years (range 10 to 65 years). Inclusion criteria: (1) patients with TLE; (2) strict history taking should be undergone before and after the PET examination and the last seizure should be kept more than 48 h before the PET examination (10); (3) able to cooperate and no contraindication for PET/CT examination; and (4) underwent brain MRI within 1 week. Exclusion criteria: (1) <10 years of age; (2) with severe systemic diseases impacting PET imaging; (3) significant neurological diseases such as syncope, conversion disorder or transient cerebral ischemia and hypoxia; (4) poor PET image quality (2); and (5) the patient’s imaging results (MRI and PET) were negative, making it impossible for the multidisciplinary team to further localize the potential epileptogenic zone. This prospective study was approved by the Research Ethics Committee of Henan Provincial People’s Hospital. A written informed consent was obtained for all adult patients and the parents of pediatric patients.

**Table 1:** Demographic and clinical data of 52 TLE.

Patient	Gender	Age (years)	VEEG	MRI	PET	EZ localization by MDT
1	M	22	R	Negative	R/MTL	R/MTL
2	M	12	R	Negative	R/MTL	R/MTL
3	F	24	L	L/LTL/FCD	L/LTL	L/LTL
4	M	18	R	L/MTL/HS	L/MTL	L/H
5	M	38	Negative	R/MTL/HA	R/MTL	R/MTL
6	F	26	L	Negative	L/MTL	L/MTL
7	M	14	L	Negative	L/MTL	L/MTL
8	F	30	L	L/LTL/FCD	L/LTL	L/LTL
9	F	20	R	L/M-LTL/FCD	L/M-LTL	L/M-LTL
10	F	46	R	R/MTL/AS	R/MTL	R/MTL
11	M	33	L	Negative	L/MTL	L/MTL
12	M	49	R	R/T/AS	R/T	R/MTL
13	F	20	R	Negative	R/LTL	R/LTL
14	M	34	L	R/MTL/HS	R/MTL	R/MTL
15	F	22	R	Negative	R/MTL	R/MTL
16	M	17	Negative	L/LTL/FCD	L/LTL	L/LTL
17	M	30	R	R/M-LTL/FCD	R/M-LTL	R/M-LTL
18	M	25	R	R/LTL/FCD	R/LTL	R/LTL
19	M	52	L	Negative	R/MTL	R/MTL
20	F	23	R	R/MTL/AS	R/MTL	R/MTL
21	M	25	Negative	R/MTL/HA	R/MTL	R/MTL
22	M	38	R	Negative	R/LTL	R/LTL
23	M	14	L	Negative	L/LTL	L/LTL
24	M	65	R	R/MTL/AS	R/MTL	R/MTL
25	F	51	R	R/MTL/HA	R/MTL	R/MTL
26	M	24	R	Negative	R/LTL	R/LTL
27	M	34	Negative	L/LTL/AC	L/LTL	L/LTL
28	M	23	L	L/MTL/MCD	L/MTL	L/MTL
29	M	25	L	L/LTL/DNET	L/LTL	L/LTL
30	F	29	R	R/MTL/HS	R/MTL	R/MTL
31	F	12	Negative	L/MTL/HA	L/MTL	L/MTL
32	F	31	R	R/LTL/CL	R/LTL	R/LTL
33	F	32	R	R/MTL/HS	R/MTL	R/MTL
34	F	21	L	L/MTL/AS	L/MTL	L/MTL
35	F	32	L	Negative	L/MTL	L/MTL
36	F	22	R	Negative	R/MTL	R/MTL
37	M	38	L	L/LTL/FCD	L/LTL	L/LTL
38	M	54	L	Negative	L/LTL	L/LTL
39	F	25	R	R/LTL/FCD	R/LTL	R/LTL
40	F	25	L	L/LTL/FCD	L/LTL	L/LTL
41	F	55	R	Negative	R/MTL	R/MTL
42	M	22	L	L/LTL/FCD	L/LTL	L/LTL
43	M	20	L	L/LTL/FCD	L/LTL	L/LTL
44	F	16	R	Negative	R/M-LTL	R/M-LTL
45	F	29	R	R/MTL/HS	R/MTL	R/MTL
46	M	10	Negative	Negative	L/LTL	L/LTL
47	M	20	L	Negative	L/LTL	L/LTL
48	F	60	R	Negative	R/MTL	R/MTL
49	F	49	L	Negative	L/LTL	L/LTL
50	F	18	L	Negative	L/T	L/T
51	M	28	L	L/LTL/FCD	L/LTL	L/LTL
52	F	34	L	L/LTL/FCD	L/LTL	L/LTL

F, female; M, male; R, right, L, left; T, temporal; HS, hippocampal sclerosis; HA, hippocampal atrophy; FCD, focal cortical dysplasia; DNET, dysembryoplastic neuroepithelial tumor; CL, cystic lesions; MCD, malformations of cortical development; AS, abnormal signal; AC, arachnoid cyst; MTL, mesial temporal lobe; LTL, lateral temporal lobe; M-LTL, mesio-lateral temporal lobe.

### Localization of EZ in TLE

All patients underwent standard pre-surgical evaluation, including clinical history and symptomatology, interictal and ictal EEG obtained during video-EEG monitoring, MRI study, PET scan and neuropsychological assessment. The localization of EZ in patients with TLE were conducted by multidisciplinary team (MDT) including senior epileptologists, clinical neurophysiologists, neuroradiologist and neurosurgeon. All patients were diagnosed with temporal lobe epilepsy based on the symptomatology, brain MRI, interictal and ictal EEG findings，and PET results. The MRI-positive group (*n* = 34) and the MRI-negative group (*n* = 18) were categorized according to the presence or absence of lesions or abnormal signal findings on MRI.

### ^18^F-FDG PET/CT imaging and MRI acquisition

Patients were fasted for at least 6 h before PET examination and blood glucose level was confirmed to be <150 mg/dL (11). Patients took antiepileptic drugs normally before and after the PET scan was performed. Intravenous ^18^F-FDG 3.7 MBq/kg (0.1 mCi/kg) was given. After the patient rested quietly for about 40 min (43.44 ± 18.04 min, mean ± SD), the early brain PET/CT examination was performed. Then the patient returned to the waiting room to rest, and the delayed PET/CT examination was performed between 2 and 3 h (160.46 ± 28.70 min) after the injection.

The acquisition of images was obtained on a Discovery VCT PET/CT scanner manufactured by GE, United States. The patient’s head was immobilized during imaging in the absence of audio and visual inputs. A spiral CT scan was first performed for attenuation correction with the following parameters: voltage 100 KV and current 240 mA. The image standard iterative method was used to obtain cross-sectional, sagittal and coronal images with a layer thickness of 3.3 mm. PET scan was then performed using the 3D-TOF acquisition method with a scan time of 10 min.

The MRI (3.0 T) acquisition coil uses a 24-channel cranial coil.The MRI scan sequence included 3D T1-weighted imaging (TR/TE = 7.2/3; matrix = 256 × 230; bandwidth = 250, layer thickness/layer spacing = 1 mm/1.5 mm), 3D T2-weighted imaging (TR/TE = 2000/288.6; matrix = 256 × 230; bandwidth = 650; layer thickness/layer spacing = 1 mm/1.5 mm), 3D T2 FLAIR (TR/TE = 6500/403.92; matrix = 256 × 230; layer thickness/layer spacing = 1 mm/1.5 mm), and 3D T2 FLAIR (TR/TE = 6500/403.92; matrix = 256 × 230; layer thickness/layer spacing = 1 mm/1.5 mm), total acquisition time is about 20 min.

### Visual analysis of PET imaging

With the knowledge of the EZ localization determined by MDT during surgical conference, the hypometabolic area of the EZ was marked with arrows on the PET images at early and delayed time points by a senior neuroradiologist. The paired PET images were then assigned to two other neuroradiologists who were blinded to the images acquired at the early and delayed time points. They visually compared the degree of asymmetry in the hypometabolic region of the two PET images and determined which image was better at identifying potential epileptogenic zone, or both images appeared to be indistinguishable. The results of 52 TLE images assessed by two physicians were categorized into three different categories: (1) early effect was better than delayed effect; (2) early effect was the same as delayed effect; and (3) delayed effect was better than early effect.

### Semi-quantitative analysis of PET imaging

Processing was conducted using an image analysis tool called uAI Research Portal (Shanghai United Imaging Intelligence Co., Ltd.) (12, 13). Step 1: Pre-processing of T1 images in the patient’s simultaneous cranial MRI, pre-processing including skull stripping, bias correction and image resampling to 1 × 1 × 1 mm^3^; Step 2: Segmentation of the T1 image. The segmentation was performed by a pre-trained cascade V-Net, which combines coarse localization and segmentation refinement. The T1 images were segmented into bits of gray matter (GM), white matter (WM) and cerebrospinal fluid (CSF), which were then segmented into 106 primary regions of interest (ROI) according to the Desikan-Killiany-Tourville [DKT; (14)] atlas. Step 3: Downsample individual DKT profiles to match PET resolution and co-align with PET. Step 4: After alignment, the mean standardized uptake value (SUVmean) was extracted for each cortical region. Both early and delayed images were extracted from the region of the EZ and the contralateral cortical region.

Asymmetry index (AI) was calculated using SUVmean in the EZ and contralateral symmetrical areas using the following formula: AI = 100 × 2 × (SUVmean_contralateral – SUVmean_EZ)/(SUVmean contralateral + SUVmean_EZ). The 100 is added here to facilitate viewing the results.

The AI value at the early time point is labeled as “AI1” and the AI value at the delayed time point is labeled as “AI2.” The difference in AI between the two time points is labeled as ΔAI. ΔAI = AI2 – AI1.

### Statistical analysis

Statistical analysis was performed using SPSS software. The kappa test was used to evaluate the consistency of the visual evaluation of the two observers. Paired *t*-tests were used to compare AIs of MRI-positive and MRI-negative temporal lobe epilepsy. The paired *t*-tests were considered statistically significant with a *p* value <0.05. For the kappa test, the consistency kappa coefficient is defined as: poor consistency = <0.20; fair agreement = 0.20–0.40; moderate agreement = 0.40–0.60; good agreement = 0.60–0.80; and very good agreement = 0.80–1.00.

## Results

### Semi-quantitative analysis of PET imaging

**Figure 1:**
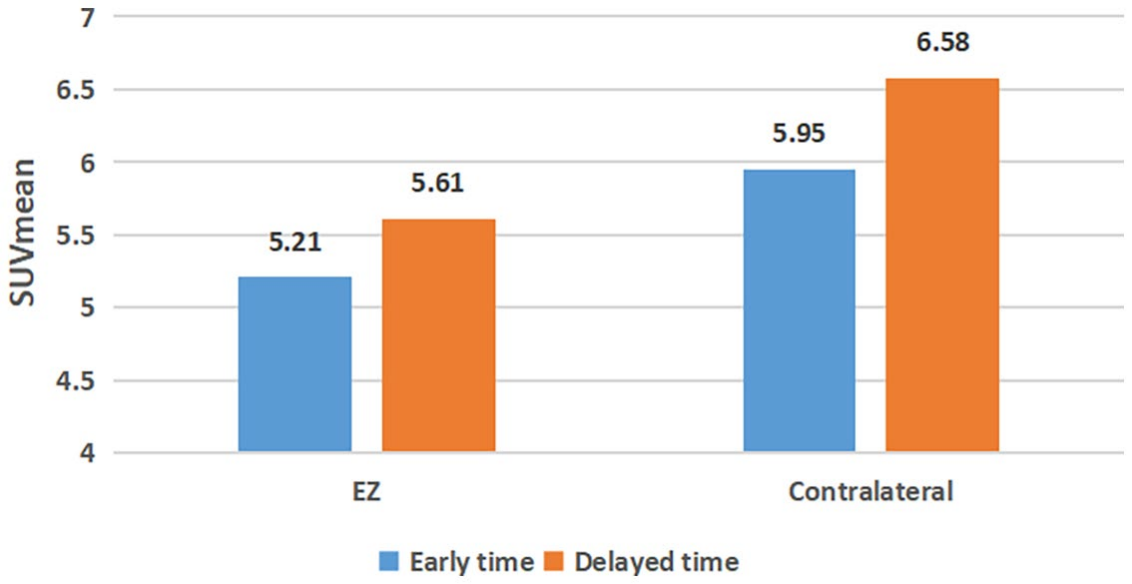
SUVmean changes of epileptogenic zone and contralateral regional at dual time points. EZ, epileptogenic zone.

Figure 1 demonstrates the changes in SUVmean of EZs and contralateral regions in 52 cases of TLE with dual time points. The SUVmean of the EZs were 5.21 ± 1.29 in the early imaging and 5.61 ± 1.52 in the delayed imaging, respectively. The SUVmean of the contralateral symmetrical regions were 5.94 ± 1.42 in the early imaging and 6.58 ± 1.63 in the delayed imaging. SUVmean increased in both the EZ and contralateral region in delayed imaging, and the increase in SUVmean was greater in the contralateral region than in the EZ.

**Figure 2:**
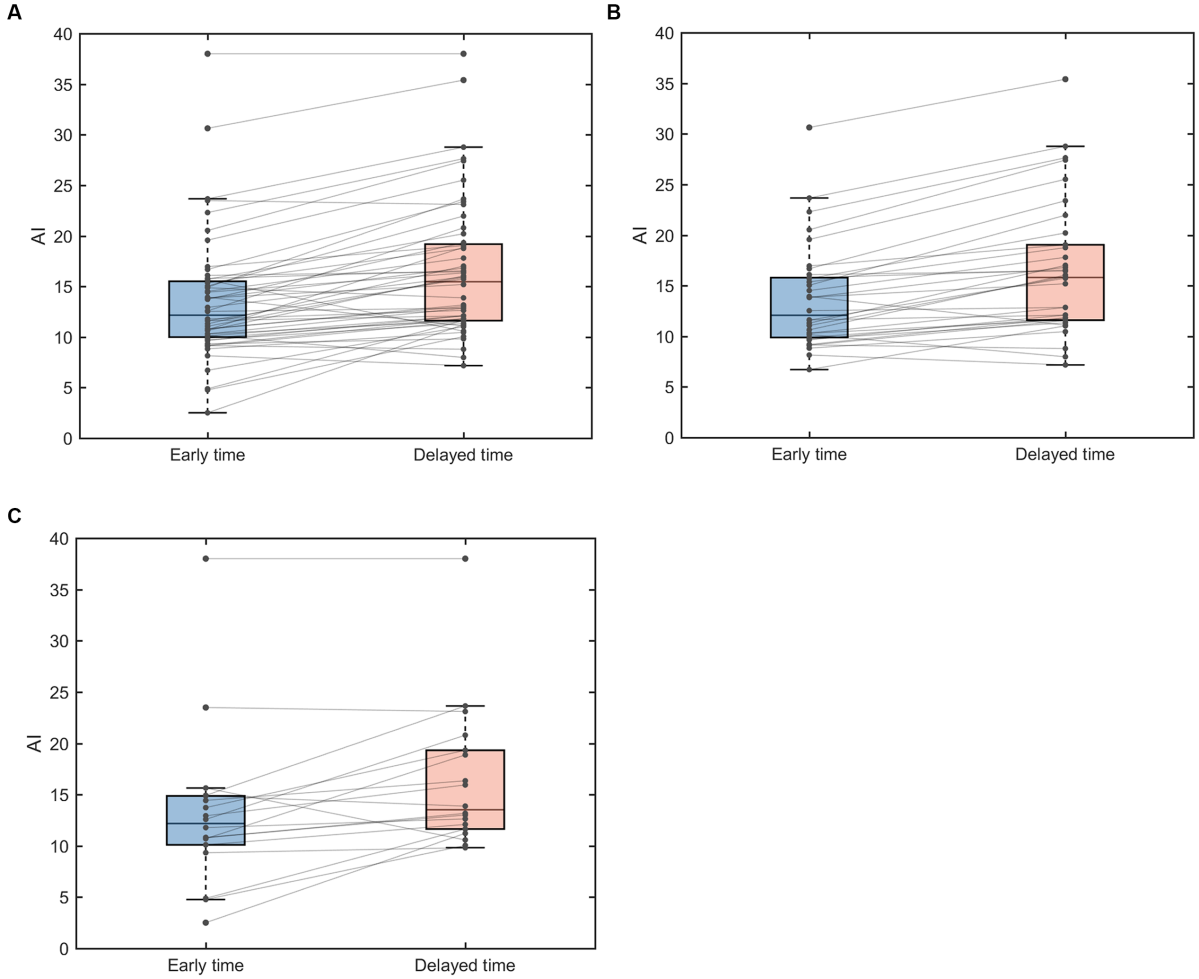
Paired box line plots of AI of EZ in TLE patients. **(A)** AI changes in 52 TLE cases. **(B)** AI changes in MRI-positive group. **(C)** MRI-negative group. AI, asymmetry index.

Paired analysis of AIs of EZ in 52 TLE patients showed that 44 patients had elevated AI values at the delayed time points (Figure 2A). AI was also elevated at the delayed time point for MRI-positive (Figure 2B) and MRI-negative (Figure 2C) groups compared to the early imaging.

The mean AI values in the 52 TLE cases were 13.47 ± 6.10 for the early imaging and 16.43 ± 6.66 for the delayed imaging, respectively, and ΔAIs was 2.95 ± 3.05. There was significant increase in AI in delayed imaging compared to the early imaging (*p* < 0.001). In the MRI positive group, the AI was 13.66 ± 5.10 for the early imaging and 16.47 ± 6.60 for the delayed imaging, and theΔAIs were 2.81 ± 2.54. The AI was significantly increased in delayed imaging compared to the early imaging (*p* < 0.001). In the MRI negative group, the AI was 13.13 ± 7.80 for the early imaging and 16.34 ± 6.97 for the delayed imaging, and the ΔAIs was 3.21 ± 3.91. The AI was also significantly increased in the delayed imaging compared to the early imaging (*p* = 0.003) (Table 2). The ΔAIs in the MRI-negative group were slightly greater than that in the MRI-positive group.

**Table 2:** AIs and ΔAIs for early and delayed time points of TLE.

	AI1	AI2	ΔAI	*P* value
TLE (52)	13.71 ± 6.10	16.43 ± 6.66	2.95 ± 3.05	0.000
MRI-positive (34, 65.38%)	13.66 ± 5.10	16.47 ± 6.60	2.81 ± 2.54	0.000
MRI-negative (18, 34.62%)	13.13 ± 7.80	16.34 ± 6.97	3.21 ± 3.91	0.003

AI1, AI at early time point; AI2, AI at delayed time point; ΔAI = AI2-AI1.

**Figure 3:**
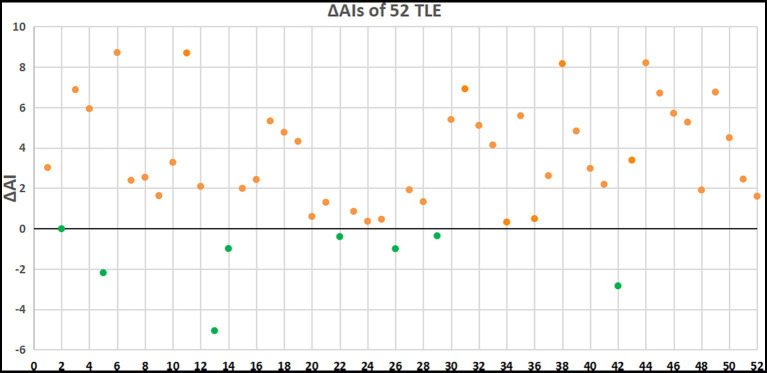
ΔAIs of 52 patients with TLE. Orange dots indicate patients with elevated AI and green dots indicate patients with decreased AI in delayed PET imaging.

Overall, 44 patients (88.61%) had an increased AI for the delayed imaging compared to the early imaging. However, 8 patients (15.38%) had a decreased AI for the delayed imaging compared to the early imaging (Figure 3). The ΔAI was −0.01, −2.19, −5.06, −0.99, −0.4, −1, −0.36, and −2.84 among the 8 patients.

### Visual analysis

Two junior neuroradiologists visually compared the effect of early and delayed images on potential EZ visualization in 52 patients with TLE. One of the physicians considered that the delayed images were visually superior to the early effect in 45 (86.54%) patients with TLE, with the early effect being the same as the delayed effect in 5 patients, and the early effect being superior to the delayed effect in 2 patients; another physician concluded that the delayed images were visually superior to the early effect in 43 patients (82.69%) with temporal lobe epilepsy, with the early effect being the same as the delayed effect in 7 patients, and the early effect was superior to the delayed effect in 2 patients. In general, two observers considered the delayed images to be better for potential EZ identification, with a good agreement between the two observers with a kappa value of 0.71.

**Figure 4:**
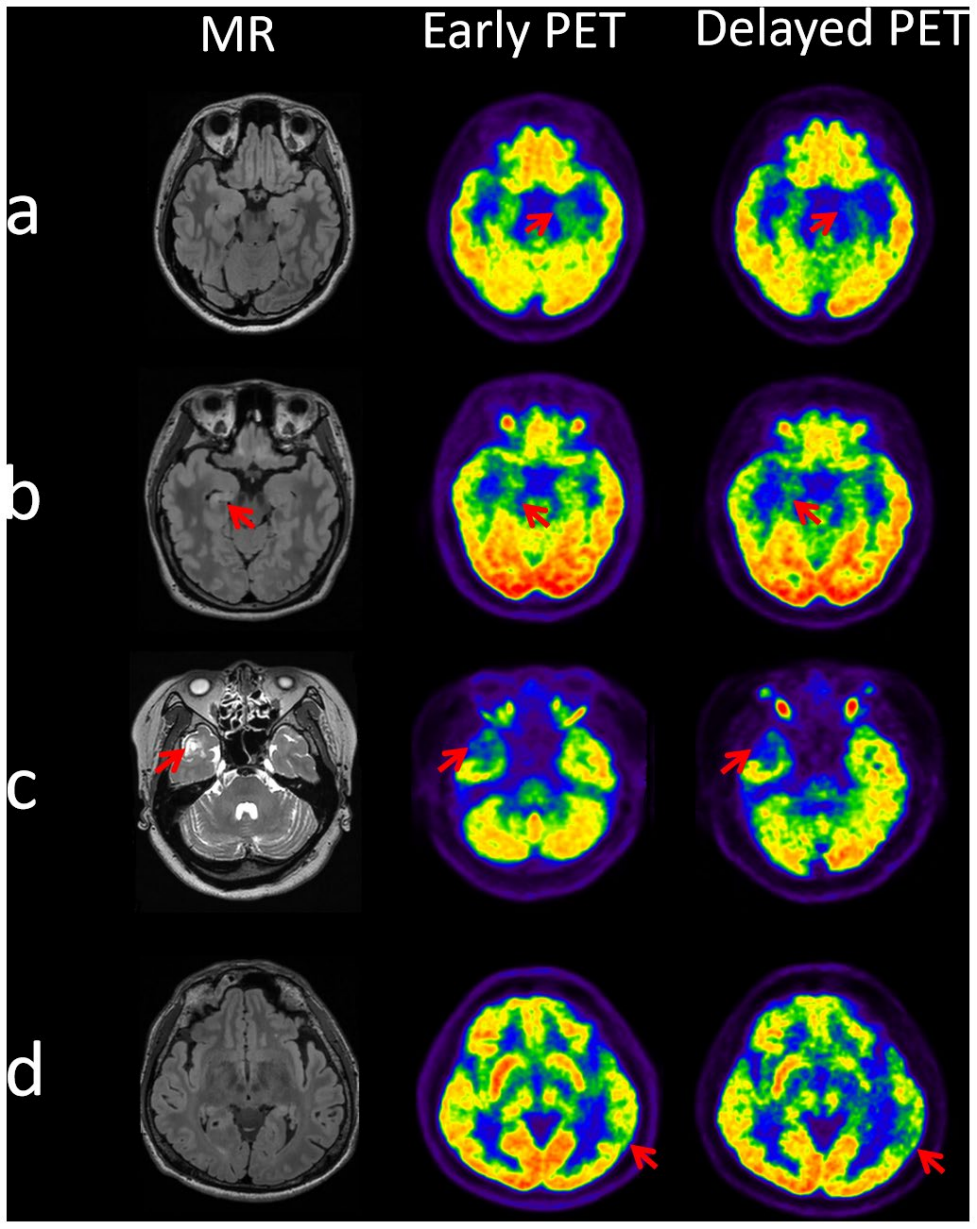
Identification of epileptogenic zones at dual time points on PET images in 4 cases. Case a (patient 6): EZ localized to the left mesial temporal lobe, two time point AIs of 14.92 (early time) and 23.64 (delayed time). Case b (patient 30): EZ localized to the right mesial temporal lobe, two time point AIs of 10.64 (early time) and 16.04 (delayed time). Case c (patient 32): EZ localized to the right lateral temporal pole, two time point AIs of 23.66 (early time) and 28.77 (delayed time). Case d (patient number 38): EZ localized to the left posterior temporal region, two time point AIs of 10.7 (early time) and 18.87 (delayed time).

Overall, as the example shown in Figure 4, the delayed images seem to be better at potential EZ identification compared to the earlier imaging.

## Discussion

In this prospective study, we performed two time point ^18^F-FDG PET studies in 52 TLE patients. Visual and semi-quantitative analysis of SUVmean and AI of the early and delayed images demonstrated that the epileptogenic focus had a more pronounced relative hypometabolism on delayed imaging than early imaging compared to contralateral regions. This is the second time that ^18^F-FDG PET two time point imaging was used to investigate the benefit of PET two phase imaging for identification of potential EZ. Our findings are in line with the study of Liu et al. using PET/MRI imaging (9).

During the processing PET data, the PET images of the patient were aligned with its own head MRI and then the SUVmean of each cortex is segmented and extracted, which is more accurate than directly aligning the PET image with the standardized template. Although the dual time point PET images are not acquired on the same machine as the patient’s MRI, the machine can correct itself during processing to achieve the best possible fit. This has been a common practice with PET and MRI co-alignment in previous studies (15).

^18^F-FDG uptake is reduced in EZ, which is likely due to the loss of neuronal cells, reduced synaptic density, and decreased neuronal cellullar activity, leading to hypometabolism in the EZs. Interictal hypometabolism in the EZ has been routinely demonstrated in PET studies (16–18). During the ictal phase, local cerebral tissue blood flow and glucose utilization are significantly increased due to excessive hyper-synchronization of epileptic firing in EZ regions. Therefore ^18^F-FDG uptake is increased, which appears as local hypermetabolism on PET imaging. It takes time for the local neuronal activity in the EZs to return to baseline after a seizure (10). Therefore, it is important that PET imaging is performed in patients who have not had a seizure for at least 48 h. In view of this phenomenon, a strict history was taken before and after the PET examination in this study to minimize the confounding effect of seizures with 48 h at the time of PET study.

Over time, the contralateral increase in ^18^F-FDG uptake was greater than the EZ, resulting in a more pronounced asymmetry index of the EZ on delayed imaging than early imaging. It is likely that normal tissue is more capable of tracer capture and retention than EZ. Over time, the slope of FDG uptake between the two time points in normal tissue contralateral to the epileptogenic zone is greater than that in the EZ. This is due to repeated abnormal discharges resulting in neuronal hypofunction in the epileptogenic region, a part of the tissue that has undergone physiological changes. Tang et al. (19)who evaluated relevant kinetic parameters in the Sokoloff model in dynamic FDG PET to understand the mechanism of epileptic hypometabolism showed that the rate at which FDG in brain tissue is phosphorylated and becomes FDG-6-P catalyzed by hexokinase at delayed time points (*k*_3_) increases, leading to an increase in FDG uptake rate (K_i_). The epileptogenic zone has a more pronounced asymmetry in the delayed PET image than in the early phase because of the greater increase in FDG uptake in normal tissue compared to the epileptogenic zone. In this regard, delayed PET imaging can is equivalent to helping to improve the accuracy of PET imaging by increasing the target-to-background change ratio (20). Generally, an AI between 10 and 15 is suggestive of EZ, and an AI >15 is diagnostic of EZ. Our data show an overall increase in AI values of delayed imaging >15, which was diagnostic of EZ. Nevertheless, AI values did not always increase in delayed imaging in our study and there was a decrease of AI in eight patients without clear explanation. Future studies with larger case volume are warranted to understand the observation.

Bilateral hypometabolism may occur in 10–20% of patients with TLE. The hypometabolism in the contralateral brain region may be due to the recurrent seizures over the course of the disease. The propagation of epileptic discharges through the corpus callosal fibers and fornix pathways inhibits the function of the contralateral region and subsequently causes hypometabolism in the contralateral temporal lobe (16). In this study, two patients presented with bilateral temporal hypometabolism, which can confound the visual and quantitative analysis in the study. In addition, it is likely that areas of hypometabolism may extend beyond the epileptogenic zone, reflecting that PET might provide lateralizing rather than localizing value (21). Our findings also showed that ΔAIs was greater in the MRI-negative group than in the MRI-positive group, suggesting that delayed PET imaging can be more valuable for the MRI negative cases. The similar results were also observed in the study using PET/MRI by Liu et al. (9).

This prospective study has several limitations. First, SEEG data or post-surgical histopathology as the gold standard for identifying EZs, particularly for mesial vs. lateral EZs, was lacking in our study. Therefore, the localization of EZ by ^18^F-FDG PET/CT hypometabolism was not validated by the intracranial EEG data and postsurgical pathology. Moreover, PET is unreliable to discriminate mesial from lateral TLE, because the extent of glucose hypometabolism during the interictal period is often larger than the extent of the EZs. First, in our study, the localization of the epileptogenic zones was decided by a multidisciplinary team in consultation with clinical symptomatology, EEG, imaging and neuropsychology. Although similar methods have been used in previous studies (22, 23), the lack of SEEG or post-surgical histopathology as the gold standard for identifying epileptogenic zone has some possibility of diagnostic error for potentially epileptogenic zone. ^18^F-FDG PET is sometimes unable to discriminate between medial TLE and lateral TLE because the extent of glucose hypometabolism changes during the interictal period is often larger than the actual extent of the EZ, which may not be accurate in patients with MRI-negative TLE (24). Second, all PET scans were performed during the interictal period. Nevertheless, none of the patients had EEG recordings after tracer injection and during the ^18^F-FDG PET scan. Therefore, subclinical seizures might have occurred during this period, which may confound the outcomes in this study (10). Finally, the delayed imaging was arbitrarily obtained between 2 and 3 h post-injection, and there is no clear evidence regarding the optimal time window for the delayed imaging. Dynamic PET acquisition can show the process of glucose uptake by the EZs, which may help find the optimal delay time, which deserves further study.

## Conclusion

Delayed ^18^F-FDG PET imaging provides significantly better identification of potential EZ in temporal lobe compared to standard early images, which can be a valuable tool in the epilepsy presurgical evaluation for patients with TLE.

## Data availability statement

The original contributions presented in the study are included in the article/supplementary material, further inquiries can be directed to the corresponding authors.

## Ethics statement

The studies involving human participants were reviewed and approved by the Ethics Committee of Henan Provincial People’s Hospital. Written informed consent to participate in this study was provided by the participants’ legal guardian/next of kin.

## Author contributions

XH, JX, JZ, and YX guided the study throughout. YaH, YC, ZR, YiH, QW, and YZ did material preparation and data collection. YaH, CF, YX, NW, TZ, YC, and YYo completed the data analysis. YYa provided technical support for data analysis. YaH and CF wrote the first draft of the manuscript. JT revised the manuscript. All authors were involved in the conception and design of the study, commented on previous versions of the manuscript, read, and approved the final manuscript.

## Funding

This study was funded by the Provincial Ministry of Medical Science and Technology Tackling Program of Henan Province (No. SBGJ202002007).

## Conflict of interest

The authors declare that the research was conducted in the absence of any commercial or financial relationships that could be construed as a potential conflict of interest.

## Publisher’s note

All claims expressed in this article are solely those of the authors and do not necessarily represent those of their affiliated organizations, or those of the publisher, the editors and the reviewers. Any product that may be evaluated in this article, or claim that may be made by its manufacturer, is not guaranteed or endorsed by the publisher.
